# Genetic Features of *mcr-1* Mediated Colistin Resistance in CMY-2-Producing *Escherichia coli* From Romanian Poultry

**DOI:** 10.3389/fmicb.2019.02267

**Published:** 2019-10-10

**Authors:** Iuliana E. Maciuca, Max L. Cummins, Andreea P. Cozma, Cristina M. Rimbu, Eleonora Guguianu, Carmen Panzaru, Monica Licker, Edit Szekely, Mirela Flonta, Steven P. Djordjevic, Dorina Timofte

**Affiliations:** ^1^Institute of Veterinary Science, University of Liverpool, Liverpool, United Kingdom; ^2^The ithree Institute, University of Technology Sydney, Sydney, NSW, Australia; ^3^Faculty of Veterinary Medicine, Ion Ionescu de la Brad, University of Agricultural Sciences and Veterinary Medicine of Iaşi, Iaşi, Romania; ^4^Microbiology Department, Grigore T. Popa University of Medicine and Pharmacy, Iaşi, Romania; ^5^Microbiology Department, Victor Babes University of Medicine and Pharmacy, Timişoara, Romania; ^6^Microbiology Department, George Emil Palade University of Medicine, Pharmacy, Science and Technology of Târgu Mures, Târgu Mureş, Romania; ^7^Clinical County Emergency Hospital Targu Mures, Târgu Mureş, Romania; ^8^Clinical Hospital of Infectious Diseases, Cluj-Napoca, Romania; ^9^Institute of Infection and Global Health, University of Liverpool, Liverpool, United Kingdom

**Keywords:** colistin-resistance, plasmid-mediated, *mcr-1* gene, poultry, humans, Romania

## Abstract

Colistin is a last resort antibiotic used for the treatment of human infections associated with carbapenemase-producing Enterobacteriales. Here, we evaluated the occurrence of *mcr-1* and *-2* plasmid-mediated colistin resistance in colistin and/or carbapenem resistant human clinical Enterobacteriales and other gram-negative bacteria (*n* = 543) as well as third generation cephalosporin-resistant (3GCR) *Escherichia coli* isolates from poultry abattoir workers (*n* = 15) and poultry fecal samples (*n* = 92) collected from two geographically separate abattoirs in Romania. which revealed that *mcr-1* was present within four sequence types (STs): ST744 (*n* = 7), ST57 (*n* = 7), ST156 (*n* = 2), and ST10 (*n* = 1). Within STs, serotypes were conserved and, notably, all except one of the *mcr-1-*positive isolates were found to exhibit fluoroquinolone-resistance (FQR) associated SNPs in both *gyrA* and *parC*. While there were variations in genotypes, all isolates belonging to ST744, ST57, and ST156 were rich in resistance determinants, carrying aminoglycoside-modifying enzymes genes, sulfonamide resistance gene *bla*_TEM–__1_ as well as *bla*_CMY–__2_ AmpC β-lactamase resistance genes. They also exhibited high similarity in carriage of virulence genes; ST10, however, only carried the *mcr-1* gene. Whole genome sequencing (WGS) analysis also revealed that although the *mcr-1* gene was identified in a diverse population of *E. coli*, two STs (ST57 and ST744) predominated and interestingly, were found in isolates across both abattoirs providing evidence for clonal transmission. Also, two main genomic contexts of *mcr-1* isolates were revealed with all ST57 isolates harboring the *mcr-1* gene between two copies of IS*Apl1* (or the Tn*6330* transposon) whilst a common *mcr-1* containing scaffold, highly similar to IncX type *mcr-1*-bearing plasmids (pWI2-mcr, Accession number: LT838201), was present among *mcr-1* isolates of varying phylogenetic backgrounds (ST10, ST744 and ST156). The high prevalence of the *mcr-1* gene in poultry *E. coli* isolates with co-resistance to cephalosporins and quinolones, in a country where antimicrobial use in food production species is poorly regulated, is concerning and the findings from this study should lead to better surveillance of antimicrobial resistance (AMR) in food-production animals in Romania.

## Introduction

The emergence and spread of carbapenem-resistance due to carbapenemase producing Enterobacteriaceae (CPE) and the lack of new antibiotic developments has led to the reintroduction of colistin for treating patients with CPE-associated infections. Therefore, colistin (also known as Polymyxin E) has been referred to as “a last-resort antimicrobial.” Increased colistin use for treatment of carbapenem resistant bacteria in human patients has led to a rise in colistin resistance due to chromosomal point mutations leading to changes of the lipid A of lipopolysaccharides, the primary target of colistin (Baron et al., 2016). In clinical human use, colistin resistance has been shown to emerge during colistin monotherapy or combination therapy (Mammina et al., 2012; Matheeussen et al., 2019). Furthermore, colistin has been used worldwide for decades in livestock, especially pig production for prevention or treatment of infections associated with Enterobacteriaceae, as well as growth promoters in countries such as China, India, Vietnam (Kempf et al., 2016). Therefore, it is unsurprising that the extensive use of colistin in food animal production has contributed to further development of colistin resistance (Kempf et al., 2016). Consequently, the discovery of plasmid mediated resistance to colistin via carriage of *mcr*-*1* in both *Escherichia coli* and *Klebsiella pneumoniae* in human and animal isolates in 2015 (Liu et al., 2016) triggered world-wide concern about the prospect of horizontal transfer of this gene amongst human and animal isolates. This discovery was followed by the investigation of numerous bacterial isolates or DNA sequence collections for the presence of the gene, which revealed *mcr-1* to be widespread in isolates from human, animal and environmental sources from countries on all five continents (Rebelo et al., 2018). This retrospective analysis of existing collections led to the identification of plasmid mediated colistin resistance in isolates collected from as far back as 1980, although most *mcr-1* carriers were identified in gram-negative isolates from 2011 to 2012 onward (Schwarz and Johnson, 2016).

Soon after the discovery of *mcr-1*, other *mcr-* variants such as *mcr-2* and *mcr-3* were identified in bovine and swine *E. coli* isolates from Belgium and China, followed by *mcr*-*4* in *E. coli* and *Salmonella* spp. from pigs in Italy, Belgium and Spain, and *mcr*-*5* which was identified in *Salmonella* Paratyphi B from poultry in Germany (Rebelo et al., 2018). Recently, *mcr-6* was described in fecal *Moraxella* spp. from healthy pigs whilst a novel colistin resistance gene (*mcr-7.1*) was described in *K. pneumoniae* isolates recovered from chickens in China (Wang X. et al., 2018; Zhang et al., 2018). Finally and very concerning, the coexistence of *mcr-8* and the carbapenemase-encoding gene *bla*_NDM_ was demonstrated in *K. pneumoniae* isolates of livestock origin in China (Wang X. et al., 2018). Worldwide, there are more reports of *mcr*-mediated resistance in livestock isolates compared to human isolates, which indicates farm animals to be a potential reservoir of plasmid mediated colistin resistance and warrants increased surveillance of animal sources as part of a process to reduce the spread of colistin-resistance. The 2014 report of the (European Centre for Disease Prevention and Control [ECDC], 2015), shows that in Romania and Greece approximately 20% of carbapenem-resistant *K. pneumoniae* isolates from blood cultures were resistant to colistin. However, there is a general lack of surveillance on antimicrobial resistance (AMR) data from animal isolates in Romania and a gap in the knowledge regarding the extent of AMR spread in livestock in this country.

The aim of this study was to investigate the prevalence of plasmid-mediated colistin resistance in a collection of cephalosporin resistant *E. coli* isolates from poultry and colistin/carbapenem resistant human clinical *E. coli* isolates from Romania, a country where antibiotic consumption in livestock is not well monitored. We also aimed to resolve the genomic structure of *mcr*-positive *E. coli* isolates and to identify whether colistin resistance spreads through clonal expansion or acquisition by different isolates. Finally, we aimed to decipher the phylogenetic relatedness and genotype characteristics of isolates obtained from poultry abattoir workers using whole genome sequencing (WGS).

## Materials and Methods

### Bacterial Isolates and Colistin Resistance Screening

**FIGURE 1:**
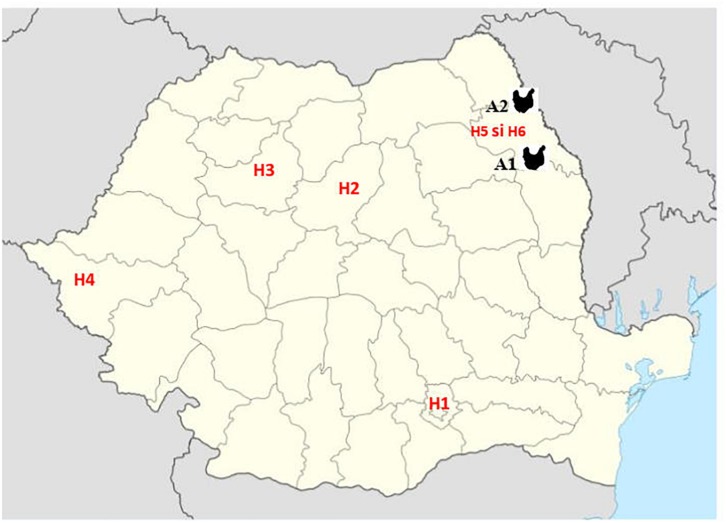
Geographic position of samples from Romanian human hospitals [H1 – Bucharest, H2 – Târgu Mureş, H3 – Cluj-Napoca, and H4 – Timişoara and Iaşi (H5 and H6)] and poultry abattoirs (A1 and A2).

Third generation cephalosporin resistant (3GCR) *E. coli* isolates obtained from 92 chickens during a previous study (Maciuca et al., 2015) and confirmed to harbor plasmid-mediated AmpC β-lactamase (pAmpC) and/or extended spectrum β-lactamase (ESBL) production were screened for colistin resistance. The isolates were obtained during October 2011–October 2012, from broiler chicken caecal samples collected from two geographically separate abattoirs (A1 and A2) in the North-East (NE) of Romania. In that study, staff working at one broiler abattoir (A1) were also screened for fecal carriage of multidrug resistant (MDR) *E. coli* to determine the potential for ESBL and AmpC producing *E. coli* transmission between the abattoir workers and the poultry they handle. Fifteen 3GCR human *E. coli* isolates obtained from abattoir workers at abattoir A1 were included for colistin-resistance screening. In addition, to determine the prevalence of *mcr*-mediated colistin resistance amongst human clinical isolates from Romanian hospitals, a total of 543 colistin and/or carbapenem resistant isolates, obtained from clinical specimens analyzed between 2014 and 2017 were included in the study. These isolates consisted of Enterobacteriales, mainly *K. pneumoniae* (*n* = 223), *E. coli* (*n* = 105), *Serratia marcescens* (*n* = 50), *Enterobacter cloacae* (*n* = 3), *Morganella morganii* (*n* = 3) and gram-negative non-fermentative bacteria (*Acinetobacter baumannii*, *n* = 28 and *Pseudomonas aeruginosa*, *n* = 131) and were obtained from four large national human hospitals (H1-Bucharest, H2-Târgu Mureş, H3-Cluj-Napoca and H4-Timişoara) and two hospitals from Iaşi, in North-East Romania (H5 and H6). The bacterial isolates from the Iaşi hospitals were from the same geographical area (same county) as the poultry isolates (Figure 1).

For the current study, colistin resistance screening was performed on MacConkey agar with colistin sulfate (Sigma-Aldrich, United Kingdom) included in the media at the (Eucast The European Committee on Antimicrobial Susceptibility Testing [EUCAST], 2018) breakpoint concentration (2 μg/ml). Where growth occurred on colistin sulfate MacConkey agar, two individual colonies (from light growth) or three individual colonies (from moderate-heavy growth) were subcultured onto 5% sheep blood agar (all media from Oxoid, Basingstoke, United Kingdom). Cell lysates from all subcultured colonies were used for DNA extraction and PCR detection of *mcr-1* and *mcr-2* gene as previously described (Liu et al., 2016; Xavier et al., 2016). The resulting amplicon DNA sequences were analyzed by Sanger sequencing and compared using BLASTn against sequences in GenBank.

### Antimicrobial Susceptibility Testing

All human clinical isolates were included in the study based on their characterization as colistin or carbapenem resistant following susceptibility testing which was performed as part of the clinical investigation in the participating hospitals. Antimicrobial susceptibility testing was performed by disk diffusion for all *mcr*-positive isolates with a panel composed of ampicillin (10 μg), amoxicillin/clavulanic acid (30 μg), cefoxitin (30 μg), cefpodoxime (10 μg), ceftiofur (30 μg), ertapenem (10 μg), nalidixic acid (30 μg), ciprofloxacin (5 μg), gentamicin (10 μg), streptomycin (10 μg), tetracycline (30 μg), trimethoprim/sulfamethoxazole 1:19 (25 μg) (all disks and media from Oxoid, United Kingdom) following EUCAST methodology and interpretation guidelines. Clinical and Laboratory Standard Institute (CLSI, 2011) interpretation guidelines were used for nalidixic acid, streptomycin, ceftiofur and tetracycline resistance. The minimal inhibitory concentration (MIC) of colistin was determined for all *mcr*-positive isolates by broth dilution. Colistin sulfate (Sigma-Aldrich Company Ltd., Dorset, United Kingdom) was used for broth microdilution and performed in accordance with the EUCAST MIC method with dilutions ranging from 0.125 μg/ml to 64 μg/ml which were performed in untreated 96-well polystyrene microplates (Greiner, Frickenhausen, Germany) according to the current recommendations of the joint CLSI-EUCAST Polymyxin Breakpoints Working Group^1^.

### Conjugation and Molecular Characterization of Isolates

To ascertain whether colistin resistance was transferable, conjugation was performed by broth mating. Conjugation assays were attempted for all isolates found to carry the *mcr-1* gene, using a streptomycin-resistant *E. coli* HB101 strain as a recipient as previously described (Oliver et al., 2002). Transconjugants were selected on nutrient agar (Oxoid, United Kingdom) supplemented with streptomycin (50 μg/ml) and colistin (2 μg/ml).

### Whole Genome Sequencing and Genome Assembly

*Escherichia coli* from poultry (*n* = 97) and human abattoir workers (*n* = 15) displaying reduced susceptibility to third generation cephalosporins (3GCR) were characterized by WGS to determine the genetic features and phylogenetic relationships shared among the collection. Specifically, we determined phylogroup, multi-locus sequence type (MLST), antibiotic resistance and virulence gene carriage and plasmid incompatibility marker carriage.

DNA was purified from *mcr-1* containing isolates using QIAGEN-QiaAmp DNA Mini kit (Qiagen, United Kingdom). DNA was quantified using a NanoDrop 2000/2000c Spectrophotometer (Thermo Fisher Scientific, United Kingdom) and standardized to 30 ng/uL before being prepared into sequencing libraries using Nextera^®^ DNA Library Preparation kits. Sequencing was performed with an Illumina HiSeq^®^ 2500, generating 150-bp paired end reads. Read quality was assessed using FastQC version 0.11.5 before *de novo* assembly using the A5 assembly pipeline version A5-miseq 20150522 (Darling et al., 2014a).

### Phylogenetic Classification, Genotyping, and Fluoroquinolone Resistance-Associated Single Nucleotide Polymorphism Analysis

Determination of e-serotype, phylogroup, MLST and carriage of genes of interest, including carriage of SNPs associated with fluoroquinolone resistance (FQR), was undertaken using the read-mapping tool ARIBA (Hunt et al., 2017). Nucleotide sequences were sourced from various public databases including VirulenceFinder, PlasmidFinder, and ResFinder from the Center for Genomic Epidemiology (Siguier et al., 2006; Zankari et al., 2012; Joensen et al., 2014) and the SRST2 serotype database (Inouye et al., 2014). Additional sequences of interest not contained therein were sourced from the NCBI nucleotide database, ISfinder (Carattoli et al., 2014) and Virulence Factor Database (Chen et al., 2005). Processing of ARIBA output files was undertaken using a custom script described in 2019 (Cummins et al., 2019). Specific versions of nucleotide databases used in the analysis, as well as workflows detailing all *in silico* analyses and the dependencies and software versions utilized to this end, are available on GitHub^2^.

### Phylogenetic Trees

Maximum-likelihood phylogenetic tree analyses were produced using the PhyloSift pipeline version 1.0.1 (Darling et al., 2014b) and FastTree 2.1.8 (Price et al., 2010), modified to resolve short branches as previously described (Wyrsch et al., 2015). Phylogenetic SNP trees were generated using Snippy version 4.3.60, Gubbins version 2.3.4 (Croucher et al., 2015), SNP-sites version 2.4.1 (Page et al., 2016) and FastTree 2.1.8. Trees were visualized using iTOL (Letunic and Bork, 2006) or the R package ggtree version 1.8.2. SNP counts were generated using SNP phylogeny^3^. Details of these workflows are also available at https://github.com/maxlcummins/Romanian-mcr-1-Escherichia-coli/.

### Global Phylogenetic Comparisons

The EnteroBase Backend Pipeline v1.1.2 (Alikhan et al., 2018) was used to determine the core-genome MLST (cgMLST) of strains found to carry *mcr-1*, according to their carriage of allelic variants of 2512 core genetic loci. Subsequently, a collection of the 116,610 strains accessible via EnteroBase (at time of writing, August 14, 2019) were compared to the *mcr-1* positive lineages under analysis, to provide lineage-specific epidemiological insights. Specifically, the most closely related strains, and the most closely related strains carrying *mcr-1*, were identified. Generally, default settings were used, except for selected reference strains for SNP analyses involving ST57, ST744, ST10, and ST156 being Liv111, Liv111M, Liv37M and Liv30MB, respectively. These analyses were facilitated by EnteroBase SNP analysis pipelines and Heirarchical Clustering of CgMLST (HierCC), which are described at https://enterobase.readthedocs.io/. See specific workflows at https://github.com/maxlcummins/Romanian-mcr-1-Escherichia-coli/.

### Determination of *mcr-1* Gene Context

Exploratory analysis of the genetic contexts of *mcr-1* in relevant assemblies was undertaken using megablast^4^ on default settings with the NCBI nucleotide collection database. Subsequent analysis involved the use of Blast Ring Image Generator (BRIG) version 0.95 (Alikhan et al., 2011) in combination with PATRIC^5^ (Wattam et al., 2017) and Snapgene^6^, to provide insight into the potential genetic context of the *mcr-1* genes. More detailed methodology is available at https://github.com/maxlcummins/Romanian-mcr-1-Escherichia-coli/.

## Results

### Bacterial Isolates, Antimicrobial Susceptibility Testing, and PCR

Initially, PCR testing identified plasmid mediated colistin resistance associated with *mcr-1* amongst 3GCR *E. coli* isolates obtained from eleven chickens (11/92, 11.9%) from both abattoirs (A1 and A2). Susceptibility testing of individual colonies obtained on the screening medium, indicated that four chickens were likely to carry two different *mcr-1* positive *E. coli* (isolates 37 and 37M; 40 and 40M; 95 and 95M; 111 and 111M), whilst *mcr-1* positive *E. coli* from another chicken showed three different antibiotypes (isolates 30, 30MA and 30MB). In total, 17 *mcr-1* positive *E. coli* isolates were obtained from 11 chickens and were characterized further. In all cases, Sanger sequencing demonstrated 100% sequence identity with *mcr-1* gene sequences deposited in the NCBI database (GenBank: KU743384.1). Eleven *mcr-1* positive *E. coli* isolates were obtained from 7 chickens from abattoir A1 and the remaining 6 isolates were obtained from 4 birds from abattoir A2. The *mcr*-2 gene was not detected in any of the poultry isolates. In addition, *mcr*-*1* and *mcr*-2 genes were not detected in any fecal isolates from abattoir workers or in any of the 543 colistin and/or carbapenem resistant human clinical isolates (both Enterobacteriales and gram-negative non-fermentative bacteria) investigated across the hospital populations. The MIC of colistin for *mcr*-*1* positive isolates varied between 4–8 μg/ml and disk diffusion susceptibility testing indicated resistance to all tested beta-lactam and quinolone antimicrobials and variable resistance to chloramphenicol and tetracyclines. However, all isolates were fully susceptible to carbapenem agents.

Conjugation experiments were successful in only one isolate (40M) where PCR identified *mcr*-*1* but not *bla*_CIT–M_ or *bla*_TEM_ genes in the transconjugant.

### WGS of the *mcr-1* Positive *E. coli* Isolates

**FIGURE 2:**
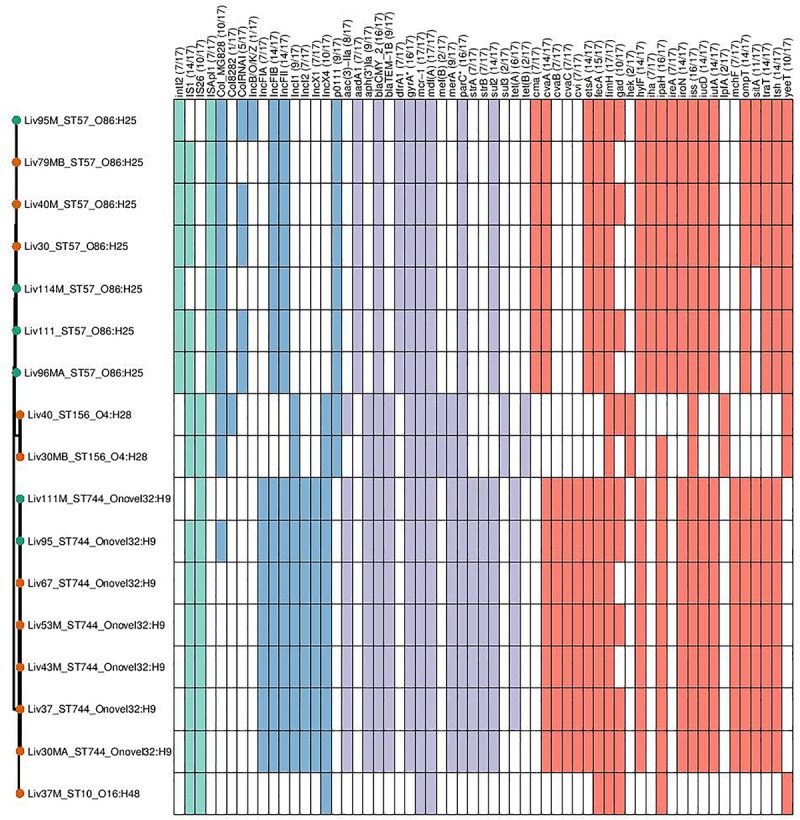
Genotypic profiles of *mcr-1* positive *E. coli* isolates shown adjacent to a Phylosift tree. The tree is midpoint rooted. Node colors on the tree are shown in red and green, corresponding to abattoir one and two, respectively. In the gene table to the right, the presence of a colored block indicates the carriage of the gene labeled atop the respective color. Teal represents genes associated with mobile genetic elements, blue indicates the presence of plasmid associated replicons, purple reflects the presence of antimicrobial resistance associated loci while red corresponds to carriage of virulence associated genes. *gyrA*^∗^; *parC*^∗^ – purple filling in such columns indicates the presence of fluoroquinolone associated SNPs.

Genomic data sets were obtained through short-read sequencing and deposited in the NCBI Sequence Read Archive (SRA) under the BioProject PRJNA560337. Individual Accession numbers can be found in Supplementary Table S1. Four sequence types were determined to carry *mcr-1*; ST744 (*n* = 7), ST57 (*n* = 7), ST156 (*n* = 2), and ST10 (*n* = 1). Carriage of genes and the sequence types, serotypes and phylogroups of *mcr-*1-positive samples can be seen in Figure 2. Within sequence type serotypes were conserved, and notably, all but one of the *mcr-1-*positive isolates, Liv37M:ST10:A:O16:H48, were found to carry FQR associated SNPs in both *gyrA* and *parC* (S83L and D87N in the former and S80I in the latter).

ST744:A:O89-O186:H9 isolates were determined to be rich in resistance gene determinants, with all isolates found to carry *aac-3-IIa*, *aph-3-Ia* aminoglycoside modifying enzymes; *strAB* streptomycin resistance gene; *sul2*-sulfonamide resistance gene; *bla*_TEM–__1_ narrow-spectrum β-lactamase resistance gene; *bla*_CMY–__2_ giving resistance to 3rd gen cephalosporins and *mcr-1* colistin resistance gene; six of seven isolates also carried the tetracycline resistance gene *tetA*. This same sequence type also consistently carried *repA* genes associated with incompatibility types IncF, IncX, and IncI, indicating the presence of three plasmid types. Carriage of extra-intestinal pathogenic *E. coli* (ExPEC) virulence associated genes (VAGs) including *cvABC*/*cvi*, *etsA*, *hylF*, *iroN*, *iss*, *iucD*/*iutA*, *ompT*, *sitA*, *traT*, and *tsh* was also widespread across this sequence type.

Within isolates of ST57:D:O86:H25, all isolates were found to carry *aac-3-IIa*, *aph-3-Ia*, *bla*_CMY–__2_, *bla*_TEM–__1_, *mcr-1*, and *sul2*. Similarly, while there was variability between samples in carriage of Col plasmid associated replicons, there was consistency in these isolates with regard to the carriage of IncF, IncX and p0111 *repA* genes. Additionally, all isolates carried a repertoire of ExPEC-associated VAGs including *cvaA*/*cma*, *etsA*, *hylF*, *iroN*, *iss*, *iucD*/*iutA*, *ompT*, *traT*, and *tsh*, while 4/7 ST57:D:O86:H25 isolates carried *sitA*.

While there were variations in genotype between ST156:A/B1:O4:H28 isolates, they exhibited high similarity in carriage of virulence, resistance and plasmid associated genes. Both isolates carried p0111, IncI and IncX *repA* genes, as well as the AMR genes *aph-3-Ia*, *bla*_CMY–__2_, *bla*_TEM–__1_, *mcr-1*, *mef*(B) encoding for macrolide resistance, *sul3* and *tet*(B). VAGs were relatively low in abundance within this lineage, with *fimH*, *hek*, *iss* and *lpfA* and *yeeT* the only VAGs detected within both isolates.

Lastly, a singular isolate of ST10:A:O16:H48, Liv37M, was found to carry *mcr-1*. This isolate also carried an IncX4 *repA* gene; however, no other plasmid replicons were detected according to ARIBA. Similarly, apart from carrying *mcr-1*, this Liv37M carried no AMR genes, and did not exhibit extensive carriage of ExPEC-associated VAGs.

### Phylogenetic Relationship of the Poultry Isolates (Abattoirs A1 and A2) and Human Isolates From Abattoir A1

**FIGURE 3:**
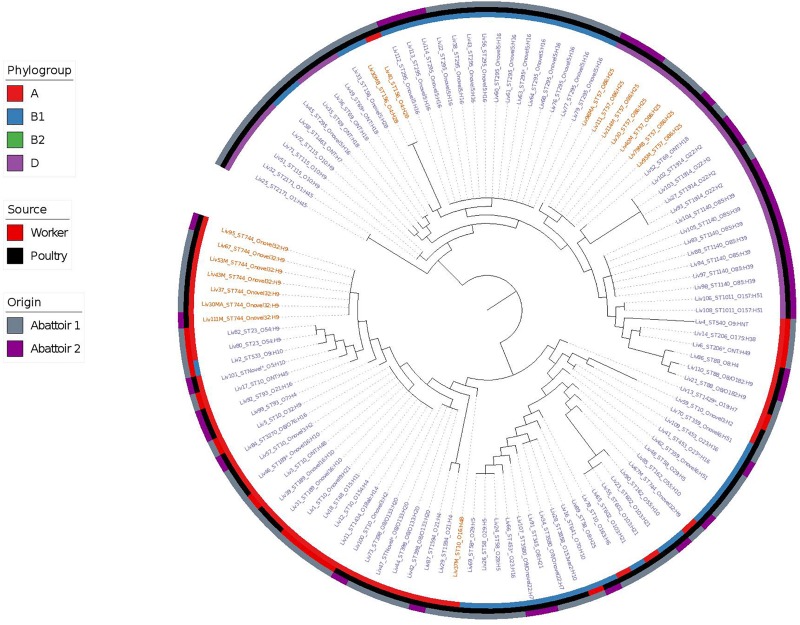
Phylogenetic relatedness of *E. coli* samples under investigation produced using Phylosift, FastTree, and iTOL. The tree is midpoint rooted. Tip labels shown in red correspond to samples that are *mcr-1* positive, while those in blue are *mcr-1* negative. Phylogroups are colored on the inner ring, while the tip labels show the sequence type and serotype of *E. coli* isolates. The middle ring shows the source of *E. coli* isolates as either of gastrointestinal origin from human abattoir workers or from poultry fecal samples, while the outer ring shows the abattoir from which the samples originate.

**FIGURE 4:**
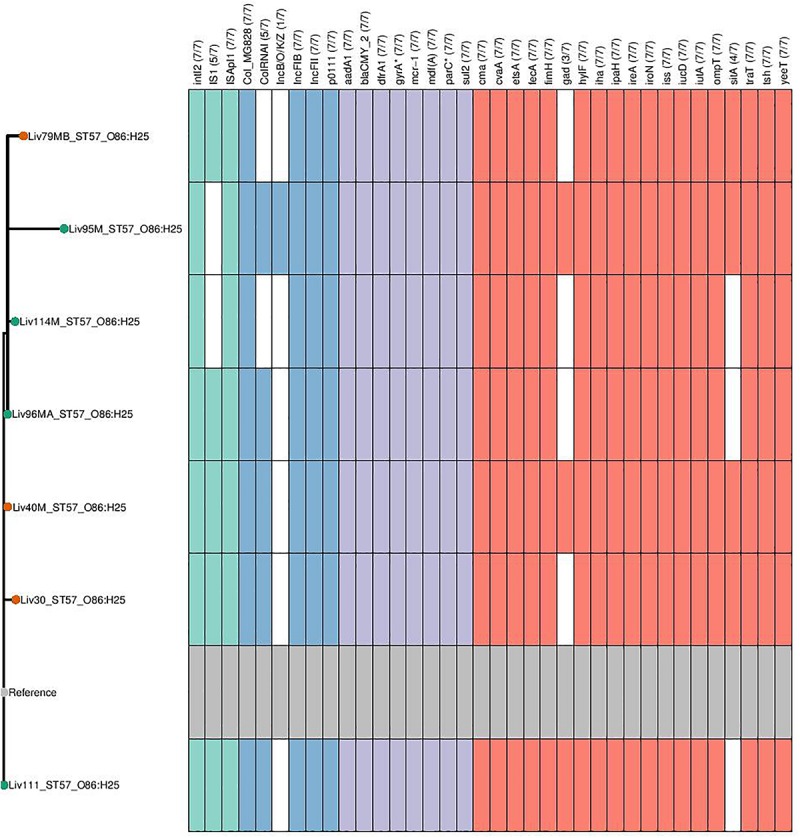
Genotypic profiles of *mcr-1* positive ST57 *E. coli* isolates shown adjacent to a SNP tree. The tree is rooted on the reference strain which is Liv111 assembled using A5. Node colors on the tree are shown in red and green, corresponding to abattoir one and two, respectively. In the gene table to the right, the presence of a colored block indicates the carriage of the gene labeled atop the respective color. Teal represents genes associated with mobile genetic elements, blue indicates the presence of plasmid associated replicons, purple reflects the presence of antimicrobial resistance associated loci while red corresponds to carriage of virulence associated genes. *gyrA*^∗^; *parC*^∗^ – purple filling in such columns indicates the presence of fluoroquinolone associated SNPs.

**FIGURE 5:**
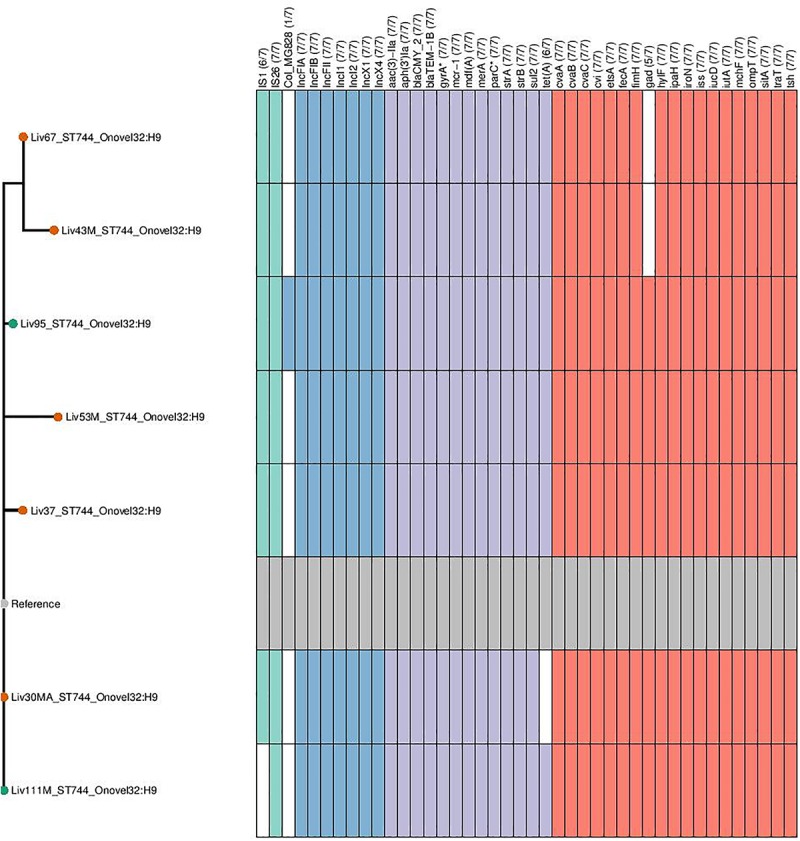
Genotypic profiles of *mcr-1* positive ST744 *E. coli* isolates shown adjacent to a SNP tree. The tree is rooted on the reference strain which is Liv111M assembled using A5. Node colors on the tree are shown in red and green, corresponding to abattoir one and two, respectively. In the gene table to the right, the presence of a colored block indicates the carriage of the gene labeled atop the respective color. Teal represents genes associated with mobile genetic elements, blue indicates the presence of plasmid associated replicons, purple reflects the presence of antimicrobial resistance associated loci while red corresponds to carriage of virulence associated genes. *gyrA*^∗^; *parC*^∗^ – purple filling in such columns indicates the presence of fluoroquinolone associated SNPs.

Phylogenetic overlap between human and poultry isolates was detected only superficially at the ST level, with ST10 isolates being common between both such sources. These samples differed in their serotypes and genotypes, however (Figure 3). In regard to the *mcr-1* positive lineages belonging to ST57 and ST744 among poultry from abattoir 1 and 2, it was found that within sequence types, fewer than 10 SNPs were identified. Additionally, samples Liv111:ST57 and Liv40M:ST57, despite being collected from different abattoirs, were found to differ by only 1 SNP. The core genomes of isolates Liv111M:ST744 and Liv30MA:ST744, which were also sourced from different abattoirs, were found to be indistinguishable by our SNP analysis [Supplementary Table S2 (ST57 SNP counts) and Supplementary Table S3 (ST744 SNP counts), respectively]. It should be noted, however, that both such pairs of isolates exhibited differences in their genotypes (Figures 4, 5). Nonetheless, the extent of sequence homology in the core genomes of both pairs of isolates, as indicated by SNP analysis, suggests they have a recent shared origin. Analysis using cgMLST was also in support of close inter-sequence type relatedness, indicating that all *mcr-1* positive strains of ST744 other than Liv37 differ only by two or less of alleles across a 2506 core genomic loci. Similarly, all but three of the *mcr-1* positive ST57 strains (Liv30, Liv40M and Liv95M) were of the same degree of relatedness, the latter of which differed by five or fewer cgMLST alleles.

### Phylogenetic Characteristics of *mcr-1* Positive *E. coli* Isolates

Overall, MLST and phylogroup (PG) typing data showed that there was evidence of clonal dissemination with some STs/PGs (i.e., ST57/D, ST744/A) being present across isolates from both abattoirs (Figure 3). In addition, MLST and PG typing has confirmed that one chicken from abattoir A1 was colonized with *mcr-1 E. coli* isolates belonging to three different STs/PGs (i.e., isolates 30, 30MA and 30MB were typed to ST57/D, ST744/A and ST156/B1) whilst four chickens (two from each abattoir) were colonized with *mcr-1* positive *E. coli* isolates belonging to two different STs and in three cases, also different phylogroups (Table 1).

**TABLE 1:** Summary of epidemiological data and genotypic typing of the 17 *mcr-1* positive *Escherichia coli* isolates obtained from 11 poultry fecal samples collected from both abattoirs (A1 and A2).

	**Sample no.**	**Time/date of isolation**	**Susceptibility profile**	**Colistin MIC μg/ml**	**ST**	**O:H**	**PG**	**IncX**	**Tn6330**
Abattoir A1	30	09/10/2010	AMP, AMC, FOX, EFT, CPD, NA, CIP	4	57	O86:H25	D	–	+
	30MA	09/10/2010	AMP, AMC, FOX, EFT, CPD, NA, CIP, CN, S	8	744	O32:H9	A	+	–
	30MB	09/10/2010	AMP, AMC, FOX, EFT, CPD, NA, CIP, STX, CN, TE	4	156	O4:28	B1	+	–
	37	03/04/2011	AMP, AMC, FOX, EFT, CPD, NA, CIP, CN, S	4	744	O32:H9	A	+	–
	37M	03/04/2011	AMP, AMC, CPD	4	10	O16:H48	A	+	–
	40	11/12/2010	AMP, AMC, FOX, EFT, CPD, NA, CIP, STX, CN, TE	4	156	O4:28	B1	+	–
	40M	11/12/2010	AMP, AMC, FOX, EFT, CPD, NA, CIP	4	57	O86:H25	D	–	+
	43M	09/10/2010	AMP, AMC, FOX, EFT, CPD, NA, CIP, CN, S	4	744	O32:H9	A	+	–
	53M	09/10/2010	AMP, AMC, FOX, EFT, CPD, NA, CIP, CN, S	4	744	O32:H9	A	+	–
	67	09/10/2010	AMP, AMC, FOX, EFT, CPD, NA, CIP, CN, S	4	744	O32:H9	A	+	–
	79MB	09/10/2010	AMP, AMC, FOX, EFT, CPD, NA, CIP, CN, S	4	57	O86:H25	D	–	+
Abattoir A2	95M	03/04/2011	AMP, AMC, FOX, EFT, CPD, NA, CIP, TE	4	57	O86:H25	D	–	+
	95	03/04/2011	AMP, AMC, FOX, EFT, CPD, NA, CIP, S, TE	4	744	O32:H9	A	+	–
	96M	05/06/2011	AMP, AMC, FOX, EFT, CPD, NA, CIP, S, TE	4	57	O86:H25	D	–	+
	111	05/06/2011	AMP, AMC, FOX, EFT, CPD, NA, CIP, C, TE	4	57	O86:H25	D	–	+
	111M	05/06/2011	AMP, AMC, FOX, EFT, CPD, NA, CIP, C, S	4	744	O32:H9	A	+	–
	114M	05/06/2011	AMP, AMC, FOX, EFT, CPD, NA, CIP	4	57	O86:H25	D	–	+

On EnteroBase, the most closely related strains to the ST744 lineage were found to differ by ≤20 of 2512 cgMLST alleles, with a single strain (SRA Accession No. ERR712576) isolated in 2013 from a hospital patient in Muenster, Germany, being found to exhibit just 19 SNPs relative to Liv111M:ST744. This strain was found by BLASTn to be *mcr-1* negative. The most closely related *mcr-1* carrying strain to Liv111M:ST744 was sourced from Italy in 2014 (additional metadata is lacking, EnteroBase Barcode: ESC_EA8845AA), which differed by 1894 SNPs and up to fifty cgMLST alleles.

Relative to Liv111:ST57, the most closely related strain (EnteroBase barcode: ESC_GA6948AA), although being of unknown origin, was found to differ by 1687 SNPs and up to 200 cgMLST alleles. This strain did not carry *mcr-1*. The most closely related *mcr-1* carrying strains were sourced from Germany in 2012 and 2011 and were sourced from the feces of a pig and a chicken, respectively (SRA Accession numbers: ERR2205952, ERR2205912) more than 6000 SNPs relative to Liv111:ST57 and differed by up to two-hundred cgMLST alleles.

Notably, three strains of ST10 (Accession numbers: SRR5830099, ERR3372571, ERR3372546) were found to be closely related to Liv37M:ST10, differing by only up to five cgMLST alleles or as many as 11 SNPs. Subsequent analysis using BLASTn indicated that these strains did not carry *mcr-1*. No single strain of ST10 that differed from Liv37M:ST10 by ≤100 cgMLST alleles was determined to carry *mcr-1*, however, the next HC of related strains HC, HC200 (strains that differ by ≤200 cgMLST alleles), consists of more than 3000 *E. coli* strains, the screening of which for *mcr-1* is beyond the scope of this project. Carriage of *mcr-1* in this larger, more distantly related cohort, is therefore unknown.

In regard to ST156, the most closely related strains on EnteroBase differed by between fifty and one-hundred cgMLST alleles, with the most phylogenetically similar of such strains, regardless of *mcr-1* carriage, determined to carry upwards of 2500 SNPs, relative to Liv30MB:ST156. The most closely related strain to carry *mcr-1* also differed by between 50 and 100 cgMLST alleles and 2667 SNPs relative to Liv30MB, and was identified in a healthy turkey from Poland in 2014 (SRA Accession No: ERS2055589).

### Investigation Into the Genetic Contexts of *mcr-1* Genes

Exploratory BLAST analysis revealed two genomic contexts of *mcr-1* within the collection. ST57 isolates carrying *mcr-1* were found to have this gene localized between two copies of IS*Apl1*, a composite transposon known as Tn*6330*. Scaffold breaks occurred within these IS elements, as is commonly the case with short-read sequencing and subsequent assembly, preventing their linkage to plasmid or chromosomally associated scaffolds, and limiting the determination of their context.

**FIGURE 6:**
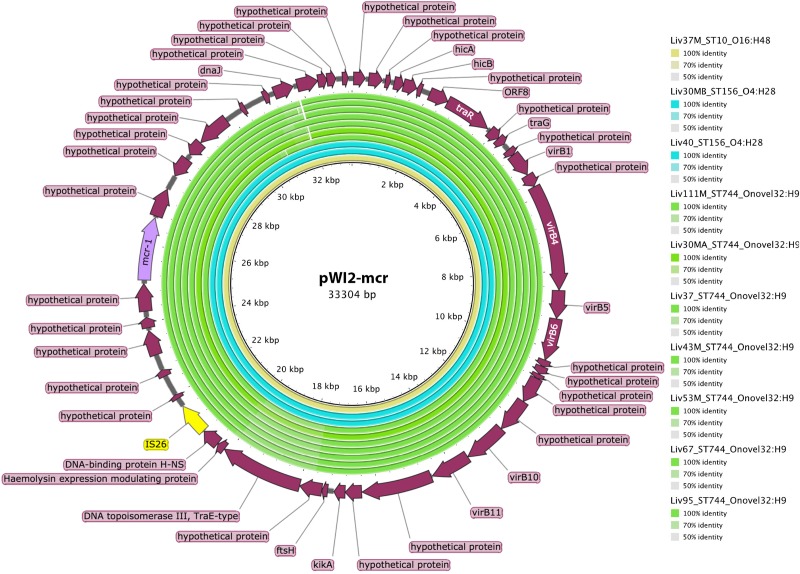
Sequence homology, shown in the inner colored rings, between *E. coli* samples under investigation and *mcr-1* bearing IncX reference plasmid pWI2-mcr (accession number LT838201). These rings are colored by sequence type, with ST10 shown in yellow, ST156 shown in blue, and ST744 shown in green. The outermost ring shows annotations for corresponding genetic loci.

However, ST744, ST156, and ST10 isolates carrying *mcr-1* did not carry this insertion sequence proximal to the *mcr-1* gene. Further analysis using BLAST revealed that the *mcr-1* containing scaffolds in these samples, some greater than 30kb in length, exhibited extensive sequence homology with publicly available IncX type *mcr-1*-bearing plasmids such as pWI2-mcr. Presence of *mcr-1* on a plasmid similar to pWI2-mcr was investigated using BRIG, as shown in Figure 6. All of these latter samples were shown to exhibit high coverage and sequence homology with the reference plasmid.

## Discussion

Here, we report a high prevalence (11.9%) of *mcr*-*1* plasmid mediated colistin resistance in commensal 3GCR AmpC producing *E. coli* from poultry sampled in two abattoirs in North-Eastern Romania in 2011/2012. Although we could not obtain data on antimicrobial usage on the farms where the samples originated, this prevalence may suggest frequent use of colistin and possibly other antimicrobials in the poultry industry in this region. Reports of the *mcr*-*1* gene in food production species are increasingly emerging worldwide, although in Europe they are still sparse and mainly originate from countries where large collections of isolates from national surveillance studies, or large datasets obtained by WGS, are readily available (Hasman et al., 2015; Kluytmans-Van Den Bergh et al., 2016; Perrin-Guyomard et al., 2016). The fact that the collection of poultry *E. coli* isolates investigated in this study is relatively small and comes from one region of Romania, makes the findings quite remarkable and could represent just a snap-shot of the general situation in the farmed poultry population in this country. Although is difficult to make direct *mcr-1* prevalence comparison between this and other studies due to different selection criteria for the isolates investigated, this is still likely to be one of the highest prevalences of *mcr*-*1* encoded colistin-resistance in commensal 3GCR *E. coli* reported for poultry in Europe and is certainly concerning. An overall prevalence of 1.2% *mcr-1* positive *E. coli* was found in a pan-European surveillance study investigating poultry isolates from 11 European countries (El Garch et al., 2018); a higher prevalence of this gene has been reported in turkey isolates from Italy (25%) as well as in poultry from China and Tunisia (Skov and Monnet, 2016; Alba et al., 2018).

Contrarily, we did not find evidence of *mcr-*mediated colistin resistance in phenotypically colistin and/or carbapenem resistant Enterobacteriales or gram-negative non-fermentative bacteria human clinical specimens from Romania. The human clinical isolates originated from six hospitals across the country, including two hospitals from the same area were the poultry samples originated. This may indicate that, at least up to 2017, when the last human isolates were analyzed, there is no evidence of over spilling of *mcr*-mediated resistance into the human population via the food chain or other routes. These findings are in agreement with other studies which show that the occurrence of *mcr* in human clinical isolates is still rare (Kluytmans-Van Den Bergh et al., 2016).

In this study, *mcr*-*1* was also not identified in 3GCR *E. coli* from abattoir workers fecal samples. However, interpretation of the role that the high prevalence of *mcr-1* identified in *E. coli* from poultry samples may have in the transmission of colistin-resistance to humans in this region or to the workers from the food chain, has to be tempered by the relatively low number of human clinical isolates and fecal samples from abattoir workers available in this study. While overlap was detected between poultry and abattoir workers at a sequence type level, this overlap was only seen in isolates belonging to ST10. This sequence type (ST10) is a globally distributed lineage associated with a wide breadth of environments including swine (Reid et al., 2017) and also human and poultry fecal carriage (Reid et al., 2019). Comparisons of the serotypes of the ST10 isolates between humans and poultry (within this collection) did not reveal any overlap, nor high phylogenetic relatedness of these isolates. However, other studies have shown that ST10 can play a major role in the dissemination of *mcr-1 E. coli* isolates from European farm animals (El Garch et al., 2017).

Most of the *mcr*-*1* positive *E. coli* identified in this study also carried plasmid mediated AmpC β-lactamase (CMY-2 type) which affords resistance to extended-spectrum cephalosporins and beta-lactam inhibitor combinations. Although *bla*_CMY–__2_ and *bla*_TEM–__1_ were not present in the single transconjugant obtained in our experiments, other similar studies have demonstrated that genes encoding colistin and ESC resistance (ESBL/AmpC) are often co-located on the same plasmids (Grami et al., 2016), which raises concerns about co-selection of these genes when either colistin or cephalosporins are used prophylactically in food production animals.

In our study, the *mcr*-1 gene was identified in a diverse population of *E. coli* where two STs (ST57 and ST744) predominated. In addition, *mcr-1* was also identified in ST57 and ST744 isolates across both abattoirs, providing evidence for clonal transmission, also evidenced by low (≤10) SNP counts observed within sequence these sequences types. For instance, ST57/D was identified in seven isolates from both abattoir A1 and A2. This ST has been previously identified in ESBL-producing *E. coli* isolates from chickens in the United Kingdom, Germany, and Canada as well as in healthy human and food-producing isolates demonstrating its potential for zoonotic transmission (Wang et al., 2013; Lemma et al., 2014). Similarly, ST744/A was identified in both abattoirs and its potential for clonal dissemination was recently demonstrated by its involvement in the epidemic spread of *mcr-3*/*bla*_CTX–M–__55_-positive *E. coli* collected in diseased veal calves from France (Haenni et al., 2018).

Investigations using EnteroBase revealed that while some strains housed in public databases that were closely related to *mcr-1* positive samples under investigation, these publically available strains were determined not to carry *mcr-1*, and that the most closely related strains to those under investigation that do carry *mcr-1* exhibit many (>1500) SNPs relative to any *mcr-1* strain identified within the collection. While only a single reference strain from each sequence type was used for this analysis, the SNP counts within STs from the two abattoirs indicate that the strains are clonal and therefore significantly higher SNP counts would not be expected if a different reference were used from within the *mcr-1* positive lineages. These data indicate that clonal expansion has taken place between sources prior to the acquisition of *mcr-1* by the Romanian strains of ST744, ST57, ST10. High resolution SNP and cgMLST analysis of publicly available genomes on EnteroBase showed no evidence of clonal associations with Liv30MB or Liv40:ST156.

The fact that *mcr-1* positive ST744 lineages differ by upwards of 1500 SNPs indicates it is likely that this sequence type has undergone multiple *mcr-1* acquisition events. This hypothesis is supported by preliminary analysis of an ST744 *mcr-1* positive *E. coli* isolate that was obtained from a human blood stream infection in Denmark (, which also carried *bla*_CMY–__2_ (amongst other resistance genes) (Hasman et al., 2015). This strain (EnteroBase Barcode: ESC_FA2130AA) exhibited 2527 SNPs compared to Liv111M, with preliminary BLAST analysis (data not shown) indicating that the *mcr-1* gene it carries is localized to an IncI plasmid similar to that of ZE36 (Accession No.: KY802014).

The ST57 lineages under investigation seemed relatively distinct from most samples present in EnteroBase, with the closest strains differing by 1687 SNPs and up to 200 cgMLST alleles. Notably, while metadata on public repositories is often lackluster, the most closely related strains (those that differed by 200 or fewer cgMLST alleles) were predominantly sourced (*n* = 25/50) from poultry-associated environments. While 7 of these correspond to ST57 isolates from the present study, removal of these strains still results in 42% (18/43) of strains deposited from seven separate Bioprojects across three countries (Australia, The United States, and Germany) being of poultry-associated origin. Note that while none of these strains other than the Romanian isolates under study carried *mcr-1*, this evidence is in support of the literature that suggests lineages of ST57 are common in poultry (Alonso et al., 2017; Cummins et al., 2019). It is also worth noting that 14% (6/43) of these strains were associated with blood or urine infections in humans, indicating the potential zoonotic role of this sample of strains, a hypothesis strengthened by the rich extraintestinal virulence gene carriage of the subset of ST57 strains in the present study. Analysis of samples on EnteroBase did not indicate that the *mcr-1* strains in the present study are part of a larger, potentially international outbreak, as the closest related strains to Liv30MB that carried *mcr-1* differed by more >6000 SNPs. As online databases are increasingly populated by whole genome sequence data, a different trend may become apparent, however.

Samples Liv30MB:ST156 and Liv40:ST156 were found to be relatively distinct at a genomic level to other isolates present in EnteroBase, differing by more than 2500 SNPs relative to the most closely related isolates, regardless of *mcr-1* carriage status. Similarly, Liv37M:ST10 also did not seem part of a larger clonal outbreak of *mcr-1* carrying strains, however, there were three stains found to differ by ≤10 SNPs and ≤5 cgMLST alleles from the strain under discussion. Unfortunately, due to poor metadata pertaining to such strains, little insight can be gained into the origins of these samples, highlighting the importance of researcher diligence in uploading metadata sufficient to facilitate epidemiological investigations; such situations are commonplace when utilizing online sequence repositories. It does, however, indicate a potential shared origin of these strains which, by metrics used in other research, may constitute clonal outbreaks (Woksepp et al., 2017). This data highlights the value of EnteroBase, and other similar tools such as BacWGSTdb (Ruan and Feng, 2016), in the undertaking of investigations involving en-masse genotypic and phylogenomic characterization; an increasingly important area of research as AMR prospects continue to worsen at a global level.

Investigations into the genetic contexts of *mcr-1* genes revealed two main genomic contexts of *mcr-1* within the collection. Firstly, all ST57 isolates harbored the *mcr-1* gene between two copies of IS*Apl1* (or the Tn*6330* transposon) which is consistent with findings from a recent study looking at the global epidemiology of *mcr-1* and which showed that a single mobilization event of the *mcr-1* gene by an IS*Apl1* transposon occurred first in 2006 (Wang R. et al., 2018). According to this study, in some lineages the flanking IS was then lost and the transposon was imported on several plasmid backgrounds which contributed to its spread. This may be the case in the second group of our *mcr-1 E. coli* isolates which belonged to the ST744, ST156 and ST10 which did not carry IS*Apl1*; instead, they harbored a common *mcr-1* containing scaffold highly similar to IncX type *mcr-1*-bearing plasmids such as pWI2-mcr, a plasmid isolated from a clinical *E. coli* isolate from France in 2016 (Beyrouthy et al., 2017). It is also worth noting that while the above IncX associated *mcr-1* isolates were of varying phylogenetic backgrounds (ST10, ST744 and ST156), those carrying Tn*6330* appear to be clonal, all being of the same sequence type (ST57) and serotype (ST57:O86H25) and being of almost identical genotype as per the virulence, plasmid and resistance genes analyzed. However, the short read sequencing performed in the current study was insufficient to localize the *Tn6330* elements to a chromosomal or extra-chromosomal context. Instead, long read sequencing will be required to gain insight in this regard. Location of *mcr-1* within a composite transposon is consequential however, as it enhances the mobility and recombinability of this gene through processes of replicative transposition and also through homologous recombination events involving the *ISApl1* genes (Snesrud et al., 2016).

It is well recognized that use of antimicrobials in food animals may contribute to development and spread of resistant organisms, particularly so in countries like Romania where antimicrobial use in both human and animals may be poorly regulated. In Romania, this situation is reflected in the high prevalence of ESBL and carbapenemase producing *E. coli* invasive human isolates reported to the EARS-Net, or by the high prevalence of CTX-M-15 ESBL enzymes in commensal *E. coli* from poultry as shown in the original study from where the current isolates were obtained (Maciuca et al., 2015). We could not obtain specific data regarding use of antimicrobials at the local farm level either for treating infections or for prophylaxis, as data on veterinary antimicrobial agents sales is completely lacking from this country. Moreover, Romania has the third highest antibiotic consumption in man in Europe whilst no data is available for consumption of antimicrobials in food-producing animals (European Centre for Disease Prevention and Control, 2014; European Medicine Agency, 2015).

The high prevalence of the *mcr-1* gene in poultry *E. coli* isolates with co-resistance to cephalosporins and quinolones, in a country which exports chicken meat to the global food market, is certainly concerning. The findings from this study suggest that rigorous surveillance of AMR in food-production animals in Romania is critical for reducing the burden of resistance genes circulating through the food chain and the associated risks for local or European (through export) food industries and markets. Finally, the recent emergence of plasmid-mediated resistance to colistin (Hasman et al., 2015; Falgenhauer et al., 2016; Hu et al., 2016; Kluytmans-Van Den Bergh et al., 2016; Liu et al., 2016; Mulvey et al., 2016; Perrin-Guyomard et al., 2016; Ruppe et al., 2016; Stoesser et al., 2016) has triggered an international review and recommendations for restrictions for colistin use in farm animals (Liu and Liu, 2018). Identifying strategies for implementing these restrictions in countries where antimicrobial use is less strictly regulated is critical for preserving the efficacy of last resort antimicrobials like colistin, for treating human and veterinary infections.

## Conclusion

The high prevalence of *mcr*-*1*plasmid mediated colistin resistance in commensal AmpC producing *E. coli* from poultry in North-Eastern Romania suggests selection of these isolates by prophylactic and/or therapeutic farm use of colistin and/or cephalosporins. This level of AMR contamination in food products will undoubtedly lead to human exposure through the food chain, representing a serious public health risk. At very least, this report provides further substantial evidence for the need to review the extensive use of colistin in food production animals in this country and also emphasizes the need to safeguard or restrict its use as a last resort antimicrobial for treating human and animal infections, especially those associated with gram-negative carbapenemase-producing bacteria.

## Data Availability Statement

All datasets generated for this study are included in the manuscript/Supplementary Files.

## Ethics Statement

This study uses strains obtained from four human hospitals in Romania. For isolates from two hospitals (Grigore T. Popa University of Medicine and Pharmacy and University of Medicine, Pharmacy, Science and Technology of Târgu Mureş), ethics approval was not required as per local procedures which state that samples/isolates resulted from the routine diagnostic process do not require further ethics approval for their use in research. The remaining two hospitals (Timişoara and Cluj-Napoca) have generic ethics approvals for research use of isolates derived from the routine diagnostic investigations: Ethics Approval No. 130/13.09.2017 issues by the Ethics Committee of Victor Babes University of Medicine and Pharmacy, Timişoara and Ethics Approval No. 10536/12.06.2018 issued by the Ethics Committee of Clinical Hospital of Infectious Diseases, Cluj-Napoca; both of these approvals include retrospective isolates obtained since 2000.

## Author Contributions

IM and AC performed the preliminary molecular testing, analyzed the data, and wrote the manuscript. MC and SD performed the whole genome sequencing analysis, analyzed the data, and wrote the manuscript. IM, CR, and EG collected and performed the phenotypic characterization of poultry isolates. CP, ML, ES, and MF collected and performed the phenotypic analysis of human clinical isolates. SD and DT planned and coordinated the study, analyzed the data, and wrote the manuscript. All authors revised and approved the final version of the manuscript.

## Conflict of Interest

The authors declare that the research was conducted in the absence of any commercial or financial relationships that could be construed as a potential conflict of interest.

## Footnotes

1 http://www.eucast.org/fileadmin/src/media/PDFs/EUCAST_files/General_documents/Recommendations_for_MIC_determination_of_colistin_March_2016.pdf

2 https://github.com/maxlcummins/Romanian-mcr-1-Escherichia-coli/

3 https://github.com/bogemad/snp_phylogeny

4 https://blast.ncbi.nlm.nih.gov/Blast.cgi

5 https://www.patricbrc.org/

6 https://www.snapgene.com
