# Supplementary material for: Genetic Features of mcr-1 Mediated Colistin Resistance in CMY-2-Producing Escherichia coli From Romanian Poultry
Source: Front Microbiol. 2019 Oct 10;10:2267. doi: 10.3389/fmicb.2019.02267 (PMC6798173; doi:10.3389/fmicb.2019.02267)
Supplement: Supplementary file 2 [file Table_2.pdf]

Supplementary Table 2 - Pairwise single nucleotide polymorphism counts between strains of ST57 strains under investigation. The reference strain utilised was Liv111.

Liv111_R1	0.0	3.0	3.0	1.0	5.0	9.0	1.0	0.0
Liv114M_R1	3.0	0.0	6.0	4.0	6.0	10.0	2.0	3.0
Liv30_R1	3.0	6.0	0.0	4.0	6.0	10.0	4.0	3.0
Liv40M_R1	1.0	4.0	4.0	0.0	6.0	10.0	2.0	1.0
Liv79MB_R1	5.0	6.0	6.0	6.0	0.0	10.0	4.0	5.0
Liv95M_R1	9.0	10.0	10.0	10.0	10.0	0.0	8.0	9.0
Liv96MA_R1	1.0	2.0	4.0	2.0	4.0	8.0	0.0	1.0
Reference	0.0	3.0	3.0	1.0	5.0	9.0	1.0	0.0
