# Supplementary material for: Genetic Features of mcr-1 Mediated Colistin Resistance in CMY-2-Producing Escherichia coli From Romanian Poultry
Source: Front Microbiol. 2019 Oct 10;10:2267. doi: 10.3389/fmicb.2019.02267 (PMC6798173; doi:10.3389/fmicb.2019.02267)
Supplement: Supplementary file 3 [file Table_3.pdf]

Supplementary Table 3 - Pairwise single nucleotide polymorphism counts between strains of ST744 strains under investigation. The reference strain utilised was Liv111M.

Liv111M_R1	0.0	0.0	2.0	5.0	4.0	2.0	1.0	0.0
Liv30MA_R1	0.0	0.0	2.0	5.0	4.0	2.0	1.0	0.0
Liv37_R1	2.0	2.0	0.0	7.0	6.0	4.0	3.0	2.0
Liv43M_R1	5.0	5.0	7.0	0.0	9.0	3.0	6.0	5.0
Liv53M_R1	4.0	4.0	6.0	9.0	0.0	6.0	5.0	4.0
Liv67_R1	2.0	2.0	4.0	3.0	6.0	0.0	3.0	2.0
Liv95_R1	1.0	1.0	3.0	6.0	5.0	3.0	0.0	1.0
Reference	0.0	0.0	2.0	5.0	4.0	2.0	1.0	0.0
